# High quality draft genomic sequence of *Flavobacterium enshiense* DK69^T^ and comparison among *Flavobacterium* genomes

**DOI:** 10.1186/s40793-015-0084-z

**Published:** 2015-11-10

**Authors:** Zhipeng Zeng, Chong Chen, Hailun Du, Gejiao Wang, Mingshun Li

**Affiliations:** State Key Laboratory of Agricultural Microbiology, College of Life Sciences and Technology, Huazhong Agricultural University, Wuhan, 430070 P. R. China

**Keywords:** *Flavobacterium*, *Flavobacterium enshiense*, Comparative genomics, Genome sequence, Pathogenicity

## Abstract

*Flavobacterium enshiense* DK69^T^ is a Gram-negative, aerobic, rod-shaped, non-motile and non-flagellated bacterium that belongs to the family *Flavobacteriaceae* in the phylum *Bacteroidetes.* The high quality draft genome of strain DK69^T^ was obtained and has a 3,375,260 bp genome size with a G + C content of 37.7 mol % and 2848 protein coding genes. In addition, we sequenced five more genomes of *Flavobacterium* type strains and performed a comparative genomic analysis among 12 *Flavobacterium* genomes. The results show some specific genes within the fish pathogenic *Flavobacterium* strains which provide information for further analysis the pathogenicity.

## Introduction

*Flavobacterium enshiense* DK69^T^ (= CCTCC AB2011144^T^ = KCTC 23775^T^) is a type strain that belongs to the genus *Flavobacterium* of the family *Flavobacteriaceae* [1]. In recent years, members of *Flavobacterium* were identified and widely distributed in soil, fresh water, marine water, sediment, microbial mat, and glaciers [2–5]. Some *Flavobacterium* strains are fish pathogens including *Flavobacterium columnare*ATCC 49512^T^ causing columnaris disease [6], *Flavobacterium psychrophilum* JIP02/86^T^ causing cold-water disease [7] and *Flavobacterium branchiophilum* FL-15^T^ causing bacterial gill disease [8].

The common characters of *Flavobacterium* strains are Gram-negative, non-spore-forming, yellow-pigmented, rod-shaped, aerobic and with a low DNA G + C content (30–41 mol %) [2–12]. The *Flavobacterium* strains contained iso-C_15:0_ as the major fatty acid, phosphatidylethanolamine as the major polar lipid and menaquinone-6 as the major respiratory quinone [9–12].

In order to provide genome information of *Flavobacterium* species, we sequenced six *Flavobacterium* strains including *F. enshiense* DK69^T^ [1]*,**Flavobacterium beibuense* F44-8^T^ [13], *Flavobacterium cauense* R2A-7^T^ [14], *Flavobacterium rivuli* WB 3.3-2^T^ [15], *Flavobacterium subsaxonicum* WB 4.1-42^T^ [15] and *Flavobacterium suncheonense* GH29-5^T^ [2]. In this study, we compared 12 genomes including the six strains that we sequenced and other six available *Flavobacterium* genomes in the NCBI, *Flavobacterium indicum* GPTSA100-9^T^ [16], *Flavobacterium frigoris* PS1^T^ [17], *Flavobacterium* sp*.* F52 [18], *Flavobacterium columnare*ATCC 49512^T^, *Flavobacterium psychrophilum* JIP02/86^T^ and *Flavobacterium branchiophilum* FL-15^T^. Here, we present the description of the non-contiguous finished genomic sequencing of *F. enshiense* DK69^T^ and the comparative genome analysis of the 12 *Flavobacterium* genomes.

## Organism information

### Classification and features

**Fig. 1:**
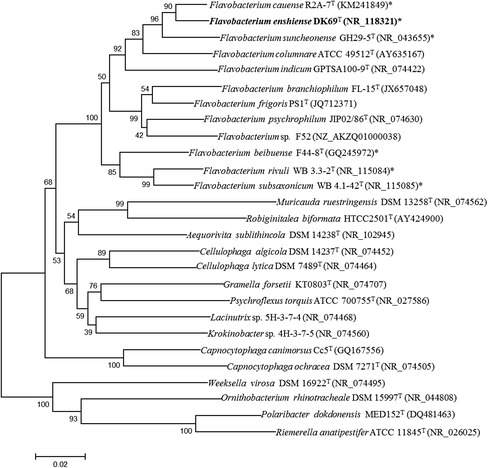
A NJ phylogenetic tree of the strains within family *Flavobacteriaceae* based on 16S rRNA gene sequence comparisons. GenBank accession numbers are shown in parentheses. The sequences were aligned using CLUSTALX, and the phylogenetic tree was obtained using MEGA 6 [19] software of neighbor-joining method [39], with the bootstrap values of 500 replicates. *represents the strains sequenced by us

**Fig. 2:**
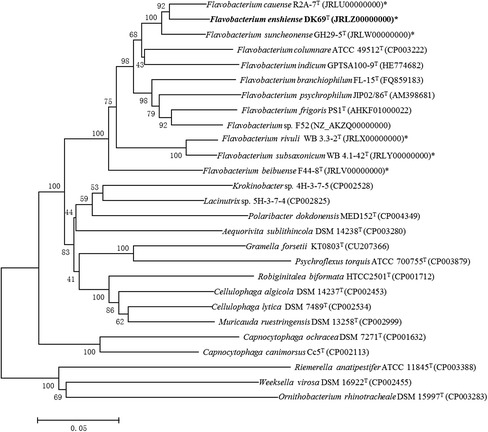
A NJ phylogenetic tree of the strains within family *Flavobacteriaceae* based on core-protein sequence comparisons. GenBank accession numbers are shown in parentheses. *represents the strains sequenced by us

*F. enshiense* DK69^T^ is a Gram-negative, strictly aerobic, yellow-pigmented rod shaped bacterium isolated from soil collected at a pharmaceutical company in Enshi, Hubei province, China. The total soil C, N, P, S and Fe concentrations were 39.83, 3.34, 0.68, 0.36, 33.80 g kg^−1^, respectively, and the pH was 6.97 [1]. A neighbor-joining phylogenetic tree based on the 16S rRNA gene sequences was built using MEGA 6 [19] and showed that strain DK69^T^ was clustered within a branch containing other species in the genus *Flavobacterium* (Fig. 1). In addition, the sequence of *F. enshiense* DK69^T^ was compared with other sequenced strains of the family *Flavobacteriaceae* use BioLinux [20], and a total of 24 core protein sequences were obtained with 50 % identity and E-value exponent of e^−10^. A phylogenetic tree based on the 24 core protein sequences of the core genome (Fig. 2) is similar to the 16S rRNA gene based tree.

**Fig. 3:**
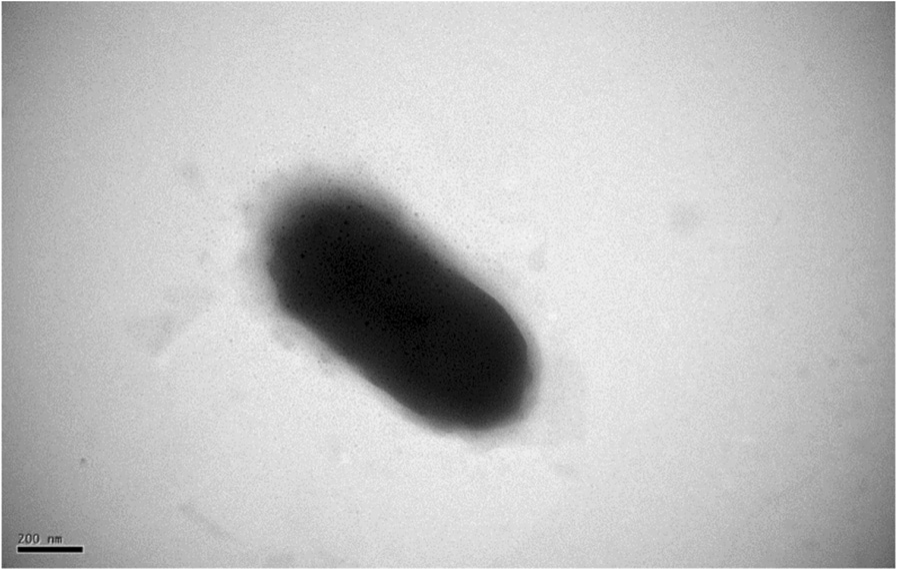
A transmission electron micrograph of *F. enshiense* DK69^T^ cells

The colonies of *F. enshiense* DK69^T^ are smooth with regular edges, circular, yellowish and about 1 mm in diameter after grown on R2A agar at 28 °C for 48 h. Growth occurs at 4–32 °C, pH 6.0–8.0 on R2A and TSA, but not on NA or LB media, and NaCl is not required [1]. Cells are non-flagellated, non-spore-forming, non-motile, rod-shaped (Fig. 3). Oxidase- and catalase- positive. The DNA G + C content is 34.4 mol% [1]. The general description of this strain is shown in Table 1.

**Table 1:** Classification and general features of *F. enshiense* DK69^T^ according to the MIGS recommendations [21]

MIGS ID	Property	Term	Evidence code
MIGS-6	Classification	Domain *Bacteria*	TAS [22]
		Phylum *Bacteroidetes*	TAS [23]
		Class *Flavobacteriia*	TAS [24]
		Order *Flavobacteriales*	TAS [24]
		Family *Flavobacteriaceae*	TAS [25]
		Genus *Flavobacterium*	TAS [5, 26]
		Species *Flavobacterium enshiense*	TAS [1]
		Type strain: DK69 ^T^ (=CCTCC AB 2011144 ^T^ = KCTC 23775 ^T^)	TAS [1]
	Gram stain	negative	TAS [1]
	Cell shape	Rod	TAS [1]
	Motility	non-motile	TAS [1]
	Sporulation	non-sporulating	TAS [1]
	Temperature range	4-32 °C	TAS [1]
	Optimum temperature	28 °C	TAS [1]
	pH range; Optimum	6.0-8.0; 7.0	TAS [1]
	Carbon source	casein, gelatin, egg yolk, tyrosine, sucrose, D-mannitol	TAS [1]
	Habitat	soil	TAS [1]
MIGS-6.3	Salinity	0 % NaCl (w/v)	TAS [1]
MIGS-22	Oxygen requirement	aerobic	TAS [1]
MIGS-15	Biotic relationship	free-living	NAS
MIGS-14	Pathogenicity	non-pathogen	NAS
MIGS-4	Geographic location	Enshi city, Hubei Province, China	TAS [1]
MIGS-5	Sample collection	2010	TAS [1]
MIGS-4.1	Latitude	not reported	
MIGS-4.2	Longitude	not reported	
MIGS-4.4	Altitude	not reported	

Evidence codes–IDA: Inferred from Direct Assay; TAS: Traceable Author Statement (i.e., a direct report exists in the literature); NAS: Non-traceable Author Statement (i.e., not directly observed for the living, isolated sample, but based on a generally accepted property for the species, or anecdotal evidence). These evidence codes are from the Gene Ontology project [27]

#### Chemotaxonomic data

The major cellular fatty acids of *F. enshiense* DK69^T^ were iso-C_15:0_, iso-C_17:1_*ω*9c, C_15:0_, iso-C_17:0_ 3-OH and iso-C_15:0_ 3-OH. The major polar lipids were phosphatidylethanolamine, one unidentified aminolipid and one unidentified lipid. *F. enshiense* DK69^T^ contained menaquinone 6 as the major quinone [1].

## Genome sequencing information

### Genome project history

Genome of *F. enshiense* DK69^T^ was sequenced by Majorbio Bio-pharm Technology Co., Ltd, Shanghai, China. The high-quality draft genome sequence was deposited in the National Center for Biotechnology Information. Contigs less than 200 bp were not included. The GenBank accession number is JRLZ00000000. The summary of the genome sequencing project information is shown in Table 2.

**Table 2:** Project information of *F. enshiense* DK69^T^

MIGS ID	Property	Term
MIGS 31	Finishing quality	High-quality draft
MIGS-28	Libraries used	Illumina Paired-End library (300 bp insert size)
MIGS 29	Sequencing platforms	Illumina Hiseq2000
MIGS 31.2	Fold coverage	487.4 x
MIGS 30	Assemblers	SOAPdenovo v1.05
MIGS 32	Gene calling method	GeneMarkS^+^
	Locus Tag	Q767
	Genbank ID	JRLZ00000000
	Genbank Date of Release	October 28, 2014
	BIOPROJECT	PRJNA221771
	Project relevance	Genome comparison
MIGS 13	Source Material Identifier	DK69^T^

### Growth conditions and genomic DNA preparation

*F. enshiense* DK69^T^ was grown on R2A medium at 28 °C for 2 d with 160 rpm shaking. Cells in late-log-phase growth were harvested and lysed by EDTA, lysozyme, and detergent treatment, followed by proteinase K and RNase digestion. The DNA was extracted and purified using the QiAamp kit according to the manufacturer’s instruction (Qiagen, Germany). The quantity of DNA was measured by the NanoDrop Spectrophotometer to ensure that the DNA concentration is greater than 20 ng/μl, then 5 μg of DNA was sent to Majorbio (Shanghai, China) for sequencing.

### Genome sequencing and assembly

The Illumina Hiseq2000 with the Paired-End library strategy was used to determine the whole-genome sequence of *F. enshiense* DK69^T^*.* TruSeq DNA Sample Preparation Kits are used to prepare DNA libraries with insert sizes of 300–500 bp for single, paired-end, and multiplexed sequencing. The protocol used 1 μg of DNA sheared by either sonication or nebulization [28]. The genome raw data of *F. enshiense* DK69^T^ generated 8,329,997 x 2 reads totaling 1,682,659,394 bp data with an average coverage of 498.4 x. Then SOAPdenovo v1.05 [29] was used to perform the following steps to assemble the sequencing data: (1) removing the adapter sequences in the reads; (2) cutting the 5’ end bases without clear A, T, C and G; (3) trimming the quality read scores lower than 20; (4) removing the reads containing more than 10 % Ns; (5) removing the reads which the length were less than 25 bp. A total of 8,217,761 x 2 high quality reads totaling 1,645,393,073 bp data with an average coverage 487.4 × was generated. The assembled sequence contained 67 scaffolds with a genome size of 3.38 Mbp.

### Genome annotation

The annotation of the genomic sequences was completed using the NCBI Prokaryotic Genome Annotation Pipeline which was combined using Best-placed reference protein set and the gene caller GeneMarkS^+^. SignalP [30] and SOSUI [31] were used to predict signal peptides and transmembrane helices. The predicted CDSs were also used to search against the Pfam protein family database [32]. The GenBank database [33] and the COG databases [34] BLASTP search were used to predict protein sequences.

## Genome properties

**Fig. 4:**
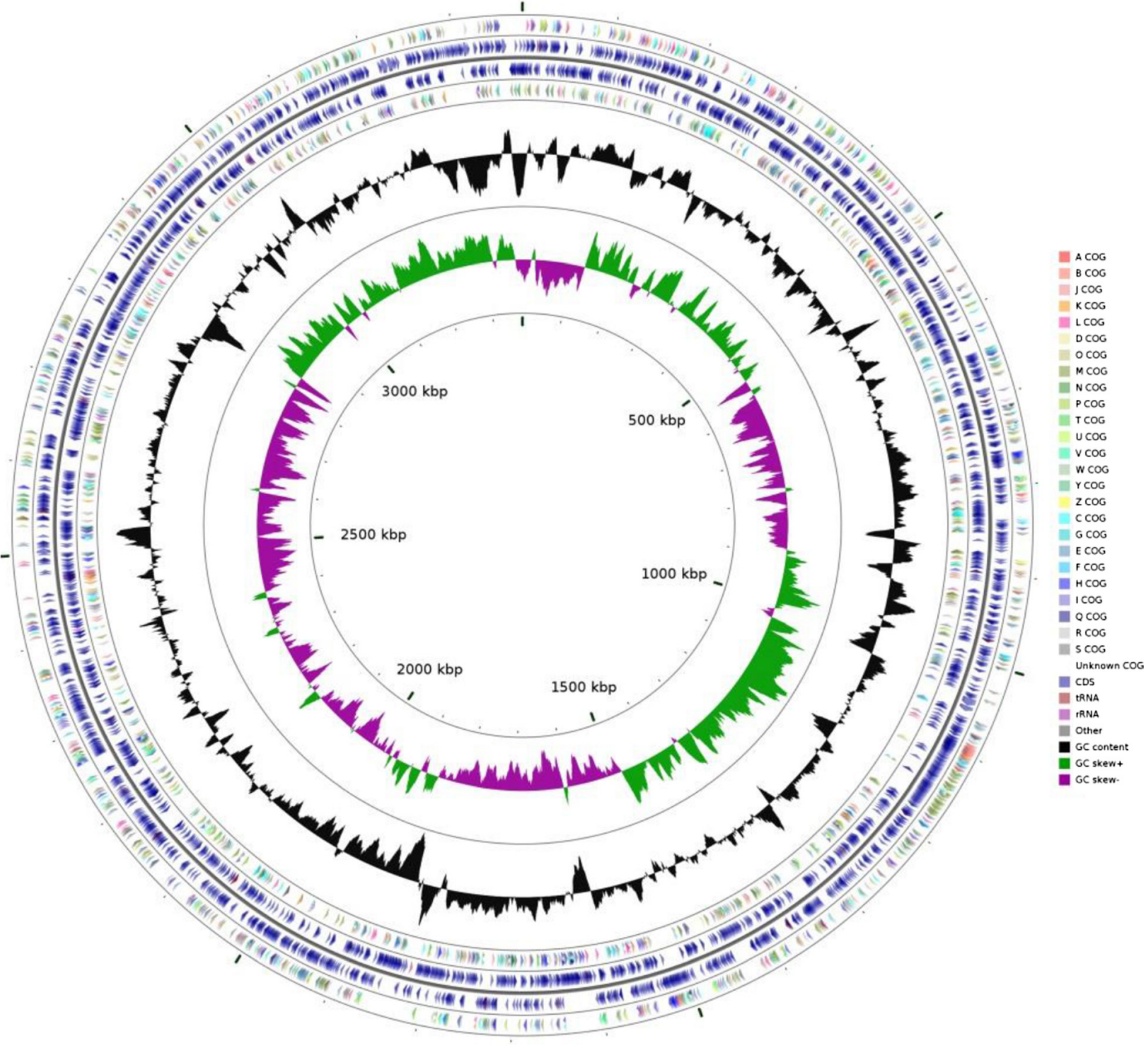
A graphical circular map of *F. enshiense* DK69^T^. From outside to inside, 1, 4 circles show forward strand or reverse strand protein-coding genes according to COG categories; 2, 3 circles show forward strand or reverse strand genes; ring 5 shows G + C% content, ring 6 shows GC skew

The genome statistics are provided in Table 3 and Fig. 4. After genome annotation, the genome of *F. enshiense* DK69^T^ was found to have a total length of 3,375,260 bp, a G + C content of 1,273,385 bp (37.7 mol %) and 74 contigs. From a total of 3,054 genes predicted, 2,848 genes are protein-coding genes, 50 are RNA genes, 57.9 % are assigned with putative functions and the remaining are annotated as hypothetical proteins or proteins of unknown functions. The distribution of genes into COGs functional categories is shown in Table 4.

**Table 3:** Genome statistics of *F. enshiense* DK69^T^

Attribute	Value	% of Total^a^
Genome size (bp)	3,375,260	100.00
DNA coding (bp)	2,808,588	83.21
DNA G + C (bp)	1,273,385	37.73
DNA scaffolds	67	-
Total genes	3054	100.00
Protein coding genes	2848	93.25
RNA genes	50	1.64
Pseudo genes	156	44.67
Genes in internal clusters	1113	3908
Genes with function prediction	1649	57.90
Genes assigned to COGs	1718	60.32
Genes with Pfam domains	2495	87.61
Genes with signal peptides	735	25.81
Genes with transmembrane helices	651	22.86
CRISPR repeats	0	-

^a^The total is based on either the size of the genome in base pairs or the total number of protein coding genes in the annotated genome

**Table 4:** Number of genes in *F. enshiense* DK69^T^ associated with general COG functional categories

Code	Value	% age^a^	Description
J	142	4.99	Translation, ribosomal structure and biogenesis
A	0	0.00	RNA processing and modification
K	76	2.67	Transcription
L	93	3.27	Replication, recombination and repair
B	1	0.04	Chromatin structure and dynamics
D	20	0.70	Cell cycle control, Cell division, chromosome partitioning
V	56	1.97	Defense mechanisms
T	67	2.35	Signal transduction mechanisms
M	176	6.18	Cell wall/membrane biogenesis
N	4	0.14	Cell motility
U	29	1.02	Intracellular trafficking and secretion
O	75	2.63	Posttranslational modification, protein turnover, chaperones
C	100	3.51	Energy production and conversion
G	54	1.90	Carbohydrate transport and metabolism
E	158	5.55	Amino acid transport and metabolism
F	60	2.11	Nucleotide transport and metabolism
H	108	3.79	Coenzyme transport and metabolism
I	69	2.42	Lipid transport and metabolism
P	81	2.84	Inorganic ion transport and metabolism
Q	39	1.37	Secondary metabolites biosynthesis, transport and catabolism
R	192	6.74	General function prediction only
S	118	4.14	Function unknown
-	1130	39.68	Not in COGs

^a^The total is based on the total number of protein coding genes in the annotated genome

## Insights from the genome sequences

### Profiles of metabolic network and pathway

**Fig. 5:**
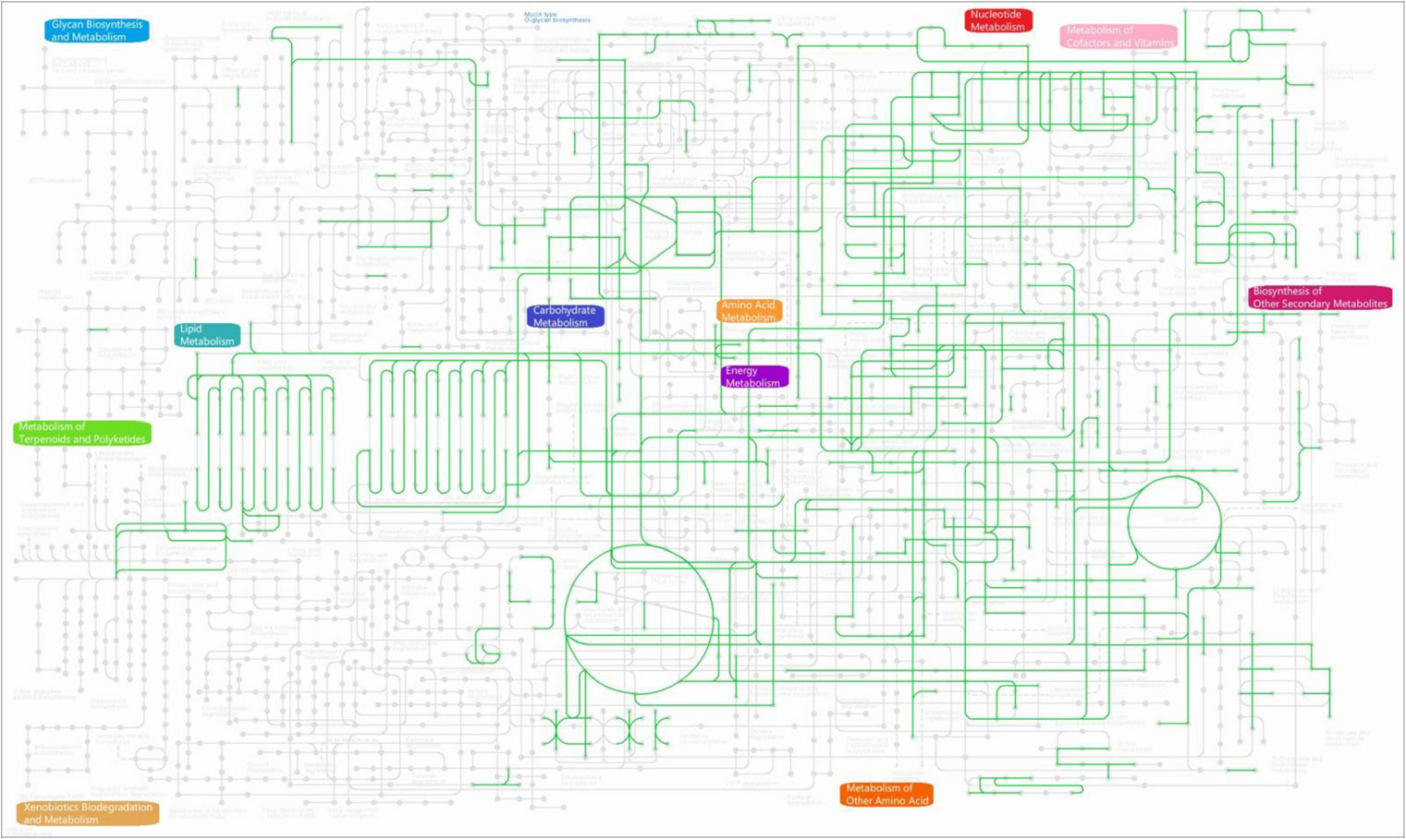
Metabolic network and pathways of *Flavobacterium enshiense* DK69^T^ as predicted using KEGG [35]. Green lines indicate pathways that are possessed by this strain

The metabolic network and pathways of *F. enshiense* DK69^T^ (Fig. 5) were predicted using the Kyoto Encyclopedia of Genes and Genomes [35]. The metabolic network showed that *F. enshiense* DK69^T^ possesses glycolysis, TCA cycle and pentose phosphate pathways and could utilize casein, tyrosine, sucrose and D-mannitol. The genome analysis results are in agreement with the phenotypes [1].

### Comparison of the 12 *Flavobacterium* genomes

**Fig. 6:**
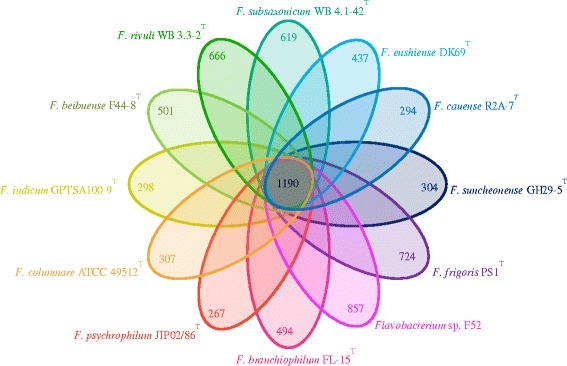
A venn diagram indicates the twelve genomes of *Flavobacterium* analyzed by OrthoMCL [36] illustrate the number of the unique proteins and the common proteins among them

The genomic information of the 12 *Flavobacterium* genomes are summarized in Table 5. OrthoMCL [36] analysis was performed to identify the set of orthologs among the 12 *Flavobacterium* genomes. *F. enshiense* DK69^T^ shared 1,190 genes with the other 11 *Flavobacterium* strains, and had 437 strain-specific genes which may contribute to the species-specific features (Fig. 6).

**Table 5:** General features of the twelve *Flavobacterium* genomes

Strains	Size (Mp)	G + C %	Total genes	CDSs	Contigs	References
*F. enshiense* DK69^T^	3.4	37.7 %	3,054	2,848	74	This study
*F. beibuense* F44-8^T^	3.8	37.7 %	3,460	3,264	61	This study
*F. cauense* R2A-7^T^	3.1	38.2 %	2,910	2,723	61	This study
*F. rivuli* WB 3.3-2^T^	4.5	39.6 %	3,975	3,691	63	This study
*F. subsaxonicum* WB 4.1-42^T^	4.6	41.6 %	4,052	3,785	80	This study
*F. suncheonense* GH29-5^T^	2.9	40.5 %	2,769	2,594	105	This study
*F. frigoris* PS1^T^	3.9	34.4 %	3,640	3,590	52	[17]
*Flavobacterium* sp. F52	5.3	34.4 %	4,601	4,549	54	[18]
*F. indicum* GPTSA100-9^T^	3.0	31.4 %	2,787	2,671	1	[16]
*F. columnare* ATCC 49512^T^	3.2	31.5 %	2,731	2,642	1	[6]
*F. psychrophilum* JIP02/86^T^	2.9	32.5 %	2,556	2,446	1	[7]
*F. branchiophilum* FL-15^T^	3.6	32.9 %	3,087	2,872	1	[8]

Three of the 12 *Flavobacterium* strains are fish pathogenic bacteria [6–8]. Using OrthoMCL [36] analysis, a total of ten proteins we found to be unique in the three fish-pathogenic species. Three of the putative proteins were reported to be related to the pathogenicity of pathogenic bacteria including polysaccharide deacetylase [37], ABC transporter ATPase and ABC transporter permease [38] (Table 6).

**Table 6:** Specific proteins of three pathogenic bacteria, *F. branchiophilum* FL-15^T^, *F. columnare* ATCC 49512^T^ *and F. psychrophilum* JIP02/86^T^

Strains	Accession	Putative protein
*F. branchiophilum* FL-15 T	WP_014083310.1	SNF2_N, HepA, PLN03142
*F. columnare* ATCC 49512 T	WP_014165166.1	
*F. psychrophilum* JIP02/86 T	WP_011962958.1	
*F. branchiophilum* FL-15 T	WP_014083635.1	hypothetical protein
*F. columnare* ATCC 49512 T	WP_014164281.1	
*F. psychrophilum* JIP02/86 T	WP_011962863.1	
*F. branchiophilum* FL-15 T	WP_014082960.1	Hexameric tyrosine-coordinated heme protein
*F. columnare* ATCC 49512 T	WP_014165359.1	
*F. psychrophilum* JIP02/86 T	WP_011963152.1	
*F. branchiophilum* FL-15 T	WP_014084059.1	polysaccharide deacetylase
*F. columnare* ATCC 49512 T	WP_014165336.1	
*F. psychrophilum* JIP02/86 T	WP_011963745.1	
*F. branchiophilum* FL-15 T	WP_014084057.1	membrane protein
*F. columnare* ATCC 49512 T	WP_014165338.1	
*F. psychrophilum* JIP02/86 T	WP_011963747.1	
*F. branchiophilum* FL-15 T	WP_014084692.1	PepSY-associated TM helix
*F. columnare* ATCC 49512 T	WP_014166184.1	
*F. psychrophilum* JIP02/86 T	WP_011963892.1	
*F. branchiophilum* FL-15 T	WP_014082991.1	S-adenosylmethionine protein
*F. columnare* ATCC 49512 T	WP_014164416.1	
*F. psychrophilum* JIP02/86 T	WP_011963983.1	
*F. branchiophilum* FL-15 T	WP_014082768.1	ABC transporter permease
*F. columnare* ATCC 49512 T	WP_014165791.1	
*F. psychrophilum* JIP02/86 T	WP_011964188.1	
*F. branchiophilum* FL-15 T	WP_014082767.1	ABC transporter ATPase
*F. columnare* ATCC 49512 T	WP_014165790.1	
*F. psychrophilum* JIP02/86 T	WP_011964189.1	
*F. branchiophilum* FL-15 T	WP_014083276.1	Transposase
*F. columnare* ATCC 49512 T	WP_014165862.1	
*F. psychrophilum* JIP02/86 T	WP_011964284.1	

## Conclusions

The genomic results of *F. enshiense* DK69^T^ and related strains reveled useful information. (1) The genome based phylogenetic analysis results is in agreement with the 16S rRNA gene based one; (2) The genomic data are correlated with some phenotypes of strain DK69^T^; (3) Compared to the three fish pathogenic *Flavobacterium**strains*, no pathogenic related genes was detected in the environmental strain DK69^T^ which indicated its non-pathogenicity; and (4) Some specific genes were found within the three fish pathogenic *Flavobacterium* strains which provides information for further analysis the pathogenicity.

## References

[1] Dong K, Chen F, Du Y, Wang G (2013). *Flavobacterium enshiense* sp. nov., isolated from soil, and emended descriptions of the genus *Flavobacterium* and *Flavobacterium cauense, Flavobacterium saliperosum* and *Flavobacterium suncheonense*. Int J Syst Evol Microbiol.

[2] Kim BY, Weon HY, Cousin S, Yoo SH, Kwon SW, Go SJ (2006). *Flavobacterium daejeonense* sp. nov. and *Flavobacterium suncheonense* sp. nov., isolated from greenhouse soils in Korea. Int J Syst Evol Microbiol.

[3] Yoon JH, Kang SJ, Oh TK (2006). *Flavobacterium soli* sp. nov., isolated from soil. Int J Syst Evol Microbiol.

[4] Yoon JH, Kang SJ, Lee JS, Oh TK (2007). *Flavobacterium terrigena* sp. nov., isolated from soil. Int J Syst Evol Microbiol.

[5] Bernardet JF, Bowman JP. Genus I. *Flavobacterium* Bergey et al. 1923. In: W. Whitman, editor. Bergey’s Manual of Systematic Bacteriology. 2nd edn, vol. 4. Baltimore: The Williams & Wilkins Co, Baltimore; 2011. p. 112–54.

[6] Tekedar HC, Karsi A, Gillaspy AF, Dyer DW, Benton NR, Zaitshik J (2012). Genome sequence of the fish pathogen *Flavobacterium columnare* ATCC 49512. J Bacteriol.

[7] Duchaud E, Boussaha M, Loux V, Bernardet JF, Michel C, Kerouault B (2007). Complete genome sequence of the fish pathogen *Flavobacterium psychrophilum*. Nat Biotechnol.

[8] Touchon M, Barbier P, Bernardet JF, Loux V, Vacherie B, Barbe V (2011). Complete genome sequence of the fish pathogen *Flavobacterium branchiophilum*. Appl Environ Microbiol.

[9] Park M, Lu S, Ryu SH, Chung BS, Park W, Kim CJ (2006). *Flavobacterium croceum* sp. nov., isolated from activated sludge. Int J Syst Evol Microbiol.

[10] Ryu SH, Park JH, Moon JC, Sung Y, Lee SS, Jeon CO (2008). *Flavobacterium resistens* sp. nov., isolated from stream sediment. Int J Syst Evol Microbiol.

[11] Sheu SY, Chiu TF, Young CC, Arun AB, Chen WM (2011). *Flavobacterium macrobrachii* sp. nov., isolated from a freshwater shrimp culture pond. Int J Syst Evol Microbiol.

[12] Xu M, Xin Y, Tian J, Dong K, Yu Y, Zhang J (2011). *Flavobacterium sinopsychrotolerans* sp. nov., isolated from a glacier. Int J Syst Evol Microbiol.

[13] Fu Y, Tang X, Lai Q, Zhang C, Zhong H, Li W (2011). *Flavobacterium beibuense* sp. nov., isolated from marine sediment. Int J Syst Evol Microbiol.

[14] Qu JH, Yuan HL, Li HF, Deng CP (2009). *Flavobacterium cauense* sp. nov., isolated from sediment of a eutrophic lake. Int J Syst Evol Microbiol.

[15] Ali Z, Cousin S, Frühling A, Brambilla E, Schumann P, Yang Y (2009). *Flavobacterium rivuli* sp. nov., *Flavobacterium subsaxonicum* sp. nov., *Flavobacterium swingsii* sp. nov. and *Flavobacterium reichenbachii* sp. nov., isolated from a hard water rivulet. Int J Syst Evol Microbiol.

[16] Barbier P, Houel A, Loux V, Poulain J, Bernardet JF, Touchon M (2012). Complete genome sequence of *Flavobacterium indicum* GPSTA100-9 T, isolated from warm spring water. J Bacteriol.

[17] Van Trappen S, Vandecandelaere I, Mergaert J, Swings J (2004). *Flavobacterium degerlachei* sp. nov., *Flavobacterium frigoris* sp. nov., and *Flavobacterium micromati* sp. nov., novel psychrophilic bacteria isolated from microbial mats in Antarctic lakes. Int J Syst Evol Microbiol.

[18] Kolton M, Green SJ, Harel YM, Sela N, Elad Y, Cytryn E (2012). Draft genome sequence of *Flavobacterium* sp. strain F52, isolated from the rhizosphere of bell pepper (*Capsicum annuum* L. cv. Maccabi). J Bacteriol.

[19] Tamura K, Stecher G, Peterson D, Filipski A, Kumar S (2013). MEGA6: molecular evolutionary genetics analysis version 6.0. Mol Biol Evol.

[20] Krampis K, Booth T, Chapman B, Tiwari B, Bicak M, Field D (2012). Cloud BioLinux: pre-configured and on-demand bioinformatics computing for the genomics community. BMC Bioinformatics.

[21] Field D, Garrity G, Gray T, Morrison N, Selengut J, Sterk P (2008). The minimum information about a genome sequence (MIGS) specification. Nat Biotechnol.

[22] Woese CR, Kandler O, Wheelis ML (1990). Towards a natural system of organisms: proposal for the domains *Archaea, Bacteria*, and *Eucarya*. Proc Natl Acad Sci U S A.

[23] Gherna R, Woese CR (1992). A partial phylogenetic analysis of the “flavobacter-bacteroides” phylum: basis for taxonomic restructuring. Syst Appl Microbiol.

[24] Berbardet JF, Krieg NR, Staley JT, Brown DR, Hedlund BP, Paster BJ (2011). Class II. *Flavobacteriia* class. nov. Bergey’s Manual of Systematic Bacteriology.

[25] Bernardet JF, Segers P, Vancanneyt M, Berthe F, Kersters K, Vandamme P (1996). Cutting a Gordian knot: emended classification and description of the genus *Flavobacterium*, emended description of the family *Flavobacteriaceae*, and proposal of *Flavobacterium hydatis* nom. nov. (Basonym, *Cytophaga aquatilis* Strohl and Tait 1978). Int J Syst Bacteriol.

[26] Bergey DH, Harrison FC, Breed RS, Hammer BW, Huntoon FM (1923). Bergey’s Manual of Determinative Bacteriology.

[27] Ashburner M, Ball CA, Blake JA, Botstein D, Butler H, Cherry JM (2000). Gene ontology: tool for the unification of biology. The Gene Ontology Con-sortium. Nat Genet.

[28] Illumina official website [www.illumina.com.cn].

[29] Xie Y, Wu G, Tang J, Luo R, Patterson J, Liu S (2014). SOAPdenovo-Trans: de novo transcriptome assembly with short RNA-Seq reads. Bioinformatics.

[30] Petersen TN, Brunak S, von Heijne G, Nielsen H (2011). SignalP 4.0: discriminating signal peptides from transmembrane regions. Nat Methods.

[31] Hirokawa T, Boon-Chieng S, Mitaku S (1998). SOSUI: classification and secondary structure prediction system for membrane proteins. Bioinformatics.

[32] Finn RD, Bateman A, Clements J, Coggill P, Eberhardt RY, Eddy SR (2014). The Pfam protein families database. Nucleic Acids Res..

[33] GenBank database. www.ncbi.nlm.nih.gov/genbank. Accessed 2 Oct 2014.

[34] Tatusov RL, Galperin MY, Natale DA, Koonin EV (2000). The COG database: a tool for genome-scale analysis of protein functions and evolution. Nucleic Acids Res.

[35] Kanehisa M, Goto S, Sato Y, Kawashima M, Furumichi M, Tanabe M (2014). Data, information, knowledge and principle: back to metabolism in KEGG. Nucleic Acids Res.

[36] Li L, Stoeckert CJ, Roos DS (2003). OrthoMCL: identification of ortholog groups for eukaryotic genomes. Genome Res.

[37] Milani CJ, Aziz RK, Locke JB, Dahesh S, Nizet V, Buchanan JT (2010). The novel polysaccharide deacetylase homologue Pdi contributes to virulence of the aquatic pathogen *Streptococcus iniae*. Microbiol.

[38] Zhang M, Han X, Liu H, Tian M, Ding C, Song J (2013). Inactivation of the ABC transporter ATPase gene in *Brucella abortus* strain 2308 attenuated the virulence of the bacteria. Vet Microbiol.

[39] Saitou N, Nei M (1987). The neighbor-joining method: a new method for reconstructing phylogenetic trees. Mol Biol Evol.

